# Beyond *BRCA1* and *BRCA2*: Deleterious Variants in DNA Repair Pathway Genes in Italian Families with Breast/Ovarian and Pancreatic Cancers

**DOI:** 10.3390/jcm9093003

**Published:** 2020-09-17

**Authors:** Aldo Germani, Simona Petrucci, Laura De Marchis, Fabio Libi, Camilla Savio, Claudio Amanti, Adriana Bonifacino, Barbara Campanella, Carlo Capalbo, Augusto Lombardi, Stefano Maggi, Mauro Mattei, Mattia Falchetto Osti, Patrizia Pellegrini, Annarita Speranza, Gianluca Stanzani, Valeria Vitale, Antonio Pizzuti, Maria Rosaria Torrisi, Maria Piane

**Affiliations:** 1 Department of Clinical and Molecular Medicine, “Sapienza” University of Rome, 00100 Rome, Italy; aldo.germani@uniroma1.it (A.G.); simona.petrucci@uniroma1.it (S.P.); patrizia.pellegrini@ospedalesantandrea.it (P.P.); mara.torrisi@uniroma1.it (M.R.T.); 2 Sant’Andrea University Hospital, 00100 Rome, Italy; Fabio.libi@ospedalesantandrea.it (F.L.); camilla.savio@gmail.com (C.S.); claudio.amanti@uniroma1.it (C.A.); adriana.bonifacino@uniroma1.it (A.B.); carlo.capalbo@uniroma1.it (C.C.); augusto.lombardi@uniroma1.it (A.L.); stefano.maggi@uniroma1.it (S.M.); mmattei@ospedalesantandrea.it (M.M.); mattiafalchetto.osti@uniroma1.it (M.F.O.); annaritasperanza@libero.it (A.S.); gstanzani@ospedalesantandrea.it (G.S.); valeria.vitale@uniroma1.it (V.V.); 3 Department of Radiological Anatomopathological, Oncological Science, “Sapienza” University of Rome, 00100 Rome, Italy; laura.demarchis@uniroma1.it; 4 Umberto I University Hospital, 00100 Rome, Italy; 5 Department of Medical and Surgical Sciences and Translational Medicine, “Sapienza” University of Rome, 00100 Rome, Italy; 6 Unit of Radiation Oncology, Sant’Andrea Hospital, Sapienza University of Rome, 00100 Rome, Italy; bcampanella@ospedalesantandrea.it; 7 Department of Molecular Medicine, “Sapienza” University of Rome, 00100 Roma, Italy; 8 Department of Experimental Medicine, “Sapienza” University of Rome, 00100 Rome, Italy; Antonio.pizzuti@uniroma1.it; 9 Clinical Genomics Unit, IRCCS Casa Sollievo della Sofferenza, 71013 San Giovanni Rotondo, Italy

**Keywords:** hereditary breast/ovarian cancer, pancreatic cancer, next-generation sequencing, gene panel, DNA repair genes

## Abstract

The 5–10% of breast/ovarian cancers (BC and OC) are inherited, and germline pathogenic (P) variants in DNA damage repair (DDR) genes *BRCA1* and *BRCA2* explain only 10–20% of these cases. Currently, new DDR genes have been related to BC/OC and to pancreatic (PC) cancers, but the prevalence of P variants remains to be explored. The purpose of this study was to investigate the spectrum and the prevalence of pathogenic variants in DDR pathway genes other than *BRCA1/2* and to correlate the genotype with the clinical phenotype. A cohort of 113 non-*BRCA* patients was analyzed by next-generation sequencing using a multigene panel of the 25 DDR pathways genes related to BC, OC, and PC. We found 43 unique variants in 18 of 25 analyzed genes, 14 classified as P/likely pathogenic (LP) and 28 as variants of uncertain significance (VUS). Deleterious variants were identified in 14% of index cases, whereas a VUS was identified in 20% of the probands. We observed a high incidence of deleterious variants in the *CHEK2* gene, and a new pathogenic variant was detected in the *RECQL* gene. These results supported the clinical utility of multigene panel to increase the detection of P/LP carriers and to identify new actionable pathogenic gene variants useful for preventive and therapeutic approaches.

## 1. Introduction

Hereditary breast, ovarian, and pancreatic cancers are associated with the presence of germline pathogenic (P) or likely pathogenic (LP) variants in the *BRCA1* and *BRCA2* genes. However, damaging mutations in these two genes justify no more than 20% of familial forms of these malignancies [1]. As the remaining 80% is still waiting for genetic diagnosis, the discovery of new genes involved in the susceptibility of hereditary cancers is under continuous investigation. The identification of pathogenic variants in other genes at both a germline and somatic is, therefore, crucial for the future of primary prevention strategies (prophylactic surgery and drug-prevention), surveillance programs, and targeted therapy. In this scenario, the research of hereditary breast/ovarian cancer susceptibility genes is crucial.

*BRCA1/2* have been primarily investigated for diagnostic purposes because their mutations show high penetrance, conferring the 5-fold higher risk of breast cancer in P/LP variant carriers compared to the general population [2].

BRCA1 and BRCA2 play a crucial role in the DNA double-strand break repair (DSBR) machinery by homologous recombination (HR). In this highly conserved mechanism, they interact with different proteins, including ATM, a master kinase acting upstream in the genome surveillance pathway, mainly activated by double-strand breaks (DSBs) [3]; MRN complex (MRE11, NBN, RAD50), able to detect DSBs [4]; CHEK2 that allows DNA repair by arresting the cell cycle at the G1/S checkpoint [5]; BARD1 and BRIP1, which interact with BRCA1 at N and C-terminal regions, respectively [6,7]; PALB2 and the paralog RAD51C and RAD51D, all involved in the BRCA complexes required for HR [8,9,10], and LKB1, encoded by *STK11*, which co-localizes with ATM and BRCA1 at the sites of the DNA damage [11]. All of these DBSR genes have been already associated with hereditary breast/ovarian and pancreatic cancers, as well as colon and gastric cancers (CC and GC) [12]. In addition, other genes involved in DNA damage repair (DDR) pathways different from HR and cell cycle control, such as *APC*, *CDH1*, *CDK4*, *CDKN2A*, *PTEN*, *SMAD4*, *TP53* [13,14,15,16,17,18], and the DNA helicase RECQL [19], have been associated with high or moderate susceptibility to familial breast cancer (BC) and other types of malignancies.

Next-generation sequencing (NGS) studies have recently demonstrated that some genes causing hereditary gastrointestinal cancer syndromes are risk factors for breast, ovarian, and pancreatic cancers. Indeed, P/LP variants in the mismatch repair (MMR) genes, such as *MSH2*, *MLH1*, *MSH6*, *PMS2*, and *EPCAM*, classically associated to hereditary colon and endometrial cancers (Lynch Syndrome), have also been identified in the breast, ovarian, biliary, and gastric tumors [20,21]. *MUTYH*, a gene involved in the DDR by base excision repair (BER) and responsible for the autosomal recessive form of familial colorectal cancer polyposis, has been recently proposed as a risk factor for breast cancer in males [22].

The identification of P/LP variants in DDR and cycle cell genes is becoming one of the main goals of the oncology clinical research. Alterations in these genes are emerging as novel targets for treatment in different cancers and, particularly, for personalized therapies. PARP (Poly(ADP-ribose) Polymerase)-inhibitors, for instance, have been introduced in the treatment of *BRCA* and, more recently, of other HR deficiency-related malignancies with encouraging results [23].

To date, however, the prevalence of germline mutations in non-*BRCA* DDR genes is partially investigated in BC, ovarian cancer (OC), and pancreatic cancer (PC), and available data about these genetic risk factors in cancer disease are still poor.

By applying NGS technologies, we analyzed 25 genes involved in DDR and in the cell cycle control in a cohort of 113 non-*BRCA* patients with personal and/or family history of BC, OC, and/or PC. This study aimed at (1) broadening the mutational spectrum and better defining the prevalence of P/LP variants in non-*BRCA* cancer-related genes, (2) evaluating the clinical utility of the multigene panel, (3) identifying novel actionable variants, and (4) improving the efficiency of clinical diagnostic tests.

## 2. Material and Methods

### 2.1. Patients

From January 2017 to December 2019, 733 unrelated patients with personal and/or familial history of BC, OC, PC attended the UOC of Medical Genetics and Advanced Cellular Diagnostics of the Department of Clinical and Molecular Medicine (Sant’Andrea University Hospital of Rome) for *BRCA1/2* molecular testing, according to the American National Comprehensive Cancer Network (NCCN) Guidelines and/or the “Regione Lazio DCA 52/201” criteria (N. U00189 del 31 May 2017). Among them, 113 probands without P/LP *BRCA* variants, who satisfied the NCCN testing criteria for the multigene panel, were selected (https://www.nccn.org/professionals/physician_gls/default.aspx). After dedicated genetic counseling, clinical data, including personal and family history, were collected by medical records and personal interviews, and the molecular analysis of 25 cancer-related genes, performed with a multigene cancer panel, was proposed. The study complied with the ethical standards of the Declaration of Helsinki and was reviewed and approved by the institutional ethics committee. Written informed consent was obtained from all participants.

### 2.2. NGS Sequencing

Genomic DNA of each patient was extracted from peripheral blood using PureLink^®^ Genomic DNA Mini Kit (Thermo Fisher Scientific, Carlsbad, CA, USA) and quantified using Qubit ds DNA HS Assay Kit on Qubit 2.0 Fluorimeter (Invitrogen, Carlsbad, CA, USA) according to the manufacturer’s instructions. By Ion Ampliseq designer software (Version 7.0, Life Technologies, Carlsbad, CA, USA; https://www.ampliseq.com/login/login.action), we designed a multigene panel, including 25 genes involved in DNA damage repair pathways as DSBR and MMR, as well as in the cell cycle control. The selection of genes was based on their association with hereditary cancer predisposition. All the selected genes (*APC, ATM, BRD1, BRIP1, CDH1, CDK4, CDKN2A, CHEK2, EPCAM, MLH1, MRE11, MSH2, MSH6, MUTYH, NBN, PALB2, PMS2, PTEN, RAD50, RAD51C, RAD51D, RECQL1, SMAD4, STK11*, and *TP53*) are indeed considered cancer-predisposing genes with high or moderate penetrance based on the relative risk for BC, OC, and other malignancies that their damaging mutations confer in carriers [2,24,25] (Table 1). The panel contains 610 primer pairs in two pools, covering the exons and exon-intron boundaries (Table S1). According to the manufacturer’s protocol, libraries were carried out by emulsion PCR using Ion PGM^TM^ (Personal Genome Machine)™ Hi-Q™ (Thermo Fisher Scientific, Carlsbad, CA, USA) View OT2 Kit (Thermo Fisher Scientific, Carlsbad, CA, USA) on Ion OneTouch 2 Instrument (Thermo Fisher Scientific, Carlsbad, CA, USA) and the Ion OneTouch ES (Enrichment System) (Thermo Fisher Scientific, Carlsbad, CA, USA) to produce high-quality Ion Sphere™ particles for use in combination with the Ion PGM™ Hi-Q™ View Sequencing Kit (Thermo Fisher Scientific, Carlsbad, CA, USA). The prepared libraries were sequenced on Ion PGM™ platform (Thermo Fisher Scientific, Carlsbad, CA, USA) using Ion 318™ Chip v2 BC. Sequencing data analysis was performed using Torrent Suite version 5.0.5 and Ion Reporter version 5.6 (Thermo Fisher Scientific, Carlsbad, CA, USA). PGM sequencing produced an average of 269,000 reads per patient, the mean read length being 198 bp. The average read depth per sample was 474 reads, with a mean percentage of reads on target of 96%. The mean percentage of regions of interest (ROI), covered at least by 100×, was 96.5% with uniformity by 97.67%. Details for each sample of the sequencing metrics are reported in Table S2. Data analysis was performed by Ion Reporter Server System v5.12 (Thermo Fisher Scientific, Carlsbad, CA, USA) and visually confirmed with the Integrative Genomics Viewer (IGV, https://igv.org/, Broad Institute and the Regents of the University of California, CA, USA). All variants reported in this paper, identified by NGS technology, were validated by Sanger sequencing.

### 2.3. Variants’ Classification

The evaluation of all identified variants was based on current evidence from the scientific literature, on gene-specific databases by LSDBs (Locus-Specific Mutation Databases) (https://grenada.lumc.nl/LSDB_list/lsdbs) and additionally by consulting ClinVar (https://www.ncbi.nlm.nih.gov/clinvar/), dbSNP (https://www.ncbi.nlm.nih.gov/snp/), Varsome (https://varsome.com), Genome Aggregation Database (gnomAD) (https://gnomad.broadinstitute.org), Exome Aggregation Consortium (ExAC). The clinical classification of the variants was carried out according to the American College of Medical Genetics and Genomics (ACMG) recommendations [31] with the 5-tier system: benign (B), likely benign (LB), variant of uncertain significance (VUS), likely pathogenic (LP), pathogenic (P). Independently, the missense prediction programs SIFT (https://sift.bii.a-star.edu.sg) Mutation Taster (http://www.mutationtaster.org), Provean (http://provean.jcvi.org/seq_submit.php), and the splice prediction tool Human Splicing Finder (HSF) (http://umd.be/Redirect.html) were queried. Genetic results were considered informative when patients carried LP or P variants, non-informative (NI) when B, LB, or VUS variants were found. Variants were reported using the Human Genome Variation Society nomenclature guidelines (https://varnomen.hgvs.org/).

### 2.4. Statistical Analysis

A comparison of demographic and clinical variables between groups was performed with unpaired *t*-test and Fisher test for continuous and categorical data, respectively. The *p*-values lower than or equal to 0.05 were considered statistically significant.

## 3. Results

From January 2017 to December 2019, a total of 113 (104 F/9 M) eligible patients, with personal and/or familial history of BC/OC and PC, who satisfied the NCCN testing criteria for the multigene panel, were included (Table 2). Among them, 87 were females with BC (77%) (fBC, mean age at onset 49.25 ± 10.35; range 30–82), including 12 cases with bilateral BC and nine with at least a second different primary cancer, seven were males with BC (6.2%) (mBC, 60.7 ± 13.2; 43–76), nine (8%) had OC (8%) (52.33 ± 14.04; 28–68), and three had PC (2.6%, 1F/2M; 65.3 ± 0.6; 65–66). The remaining seven probands (6.2%), all women, had other types of cancers (melanoma, Mullerian sarcoma, thyroid papilloma) and/or malignancies in their relatives. Overall, 107/113 cases (94.7%) referred a positive family history for BC, OC, prostatic cancer (PrC), PC, and/or other types of malignancies in one or more relatives (Table 2).

### 3.1. Multigene Panel Results

**Figure 1:**
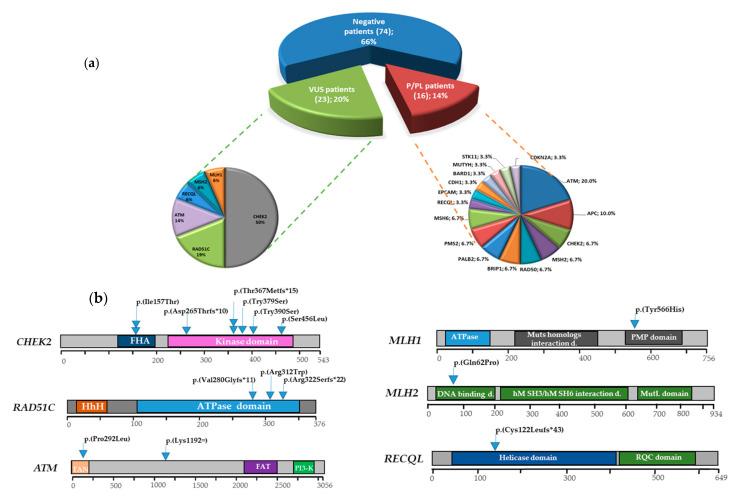
Distribution of patients with and without P/LP (P, pathogenic, LP, likely pathogenic variants). (**a**) Sixteen out of 113 patients were identified with P/LP variants. (**b**) Schematic representation of the *CHEK2*, *RAD51C*, *ATM*, *MLH2*, *MLH1*, *RECQL* genes and positions of identified P/LP variants. FHA: Fork-head-associated domain; HhH, helix-hairpin-helix motif; TAN, Tel1/ATM N-terminal motif; FAT, FRAP-ATM-TRRAP domain; PIKK or PI3K, phosphatidylinositol 3-kinase-related kinase domain; PMP, PMS2/MLH3/PMS1 interaction domain; Muts, DNA-binding domain of DNA mismatch repair; RQC, RecQ carboxy-terminal domain.

In 16/113 BRCA1/2-negative index cases (14%), we identified LP/P variants, whereas 23 probands were the carrier of VUS (20%). The remaining 74 (66%) had no damaging mutations or VUS (Figure 1a). Excluding the B/LB variants, we identified 43 unique variants in 18 of 25 analyzed genes. They included 33 (77%) missense, six (14%) intronic, two (5%) frameshift, one splicing mutation (2%), and one synonym variants (2%). Fourteen unique variants (33.3%), found in five genes involved in DSBR-HR, MMR, cell cycle regulation, and in the DNA repair helicase gene RECQL, were classified as P/LP. In particular, six were detected in the CHEK2 gene, three in RAD51C, two in ATM, while single variants P/LP were detected in RECQL, MLH1, and MSH2 genes. The pathogenic variant c.363_364del in the RECQL gene has not been previously reported. The remaining 29 identified variants were VUS (67%), four of which, found in RAD50, BRIP1, and ATM, were new (Figure 1a,b, Table 3). All the 16 probands had malignancies: 10 had BC, two OC, one PC, and the remaining three melanoma, Mullerian sarcoma, and thyroid papilloma, respectively. No asymptomatic probands were found with damaging mutations. Clinical features and family history of the patients carrying pathogenic variants identified in this study are summarized in Table 4.

### 3.2. DSBs-HR Genes Variants and Related Phenotypes

#### 3.2.1. *ATM*

The LP *ATM* variant c.875C>T was indeed detected in a 44-year-old woman (P29) with a well-differentiated and hormone-responsive in situ ductal carcinoma (DCIS) diagnosed at 43 years, who had removed a melanoma a year before. Melanoma, osteosarcoma, head-neck, brain, and uterine cancers were referred in her paternal relatives (Figure S1c). NGS analysis in this patient also detected a VUS c.377C>T, p.(Pro126Leu) in *RECQL*. The *ATM* c.875C>T, p.(Pro292Leu), is a rare variant detected in 0.01% of the general population (ExAC source). Although located in a protein domain with unknown function, it has been described in a compound heterozygous state in AT cases [32,33]. Functional studies have demonstrated a low level of the protein, absent or reduced functional kinase activity, and high radio-sensitivity after ionizing radiation exposure of cells carrying this mutation [34,35].

**Figure 2:**
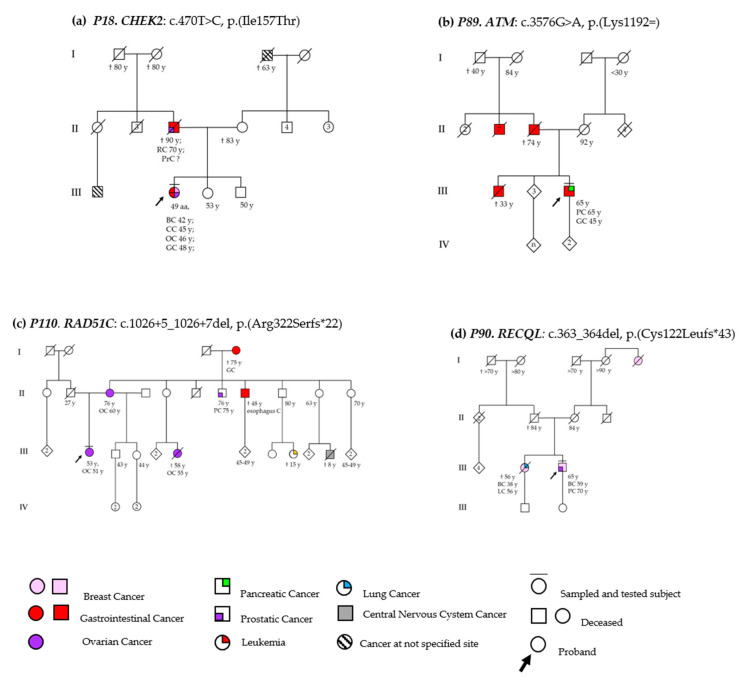
Pedigrees of four families with P/LP variants. Individuals with any cancer are shown as Figure 2. (**a**) *CHEK2* family, (**b**) *ATM* family, (**c**) *RAD51C* family, (**d**) *RECQL* family. The tested subject is indicated with a horizontal line above the circle or square. BC, breast cancer; OC, ovarian cancer; GC, gastric cancer; PC, pancreatic cancer; CC, colon cancer; RC, colonrectal cancer; PrC, prostatic cancer; LC, lung cancer.

The patient (P89) carrying the pathogenic *ATM* variant c.3576G>A had pancreatic cancer at 66 years of age. Twenty-one years earlier, he underwent surgery for a GC. In addition, his brother, his father, and seven of his paternal uncles deceased by GC (Figure 2b). The c.3576G>A substitution is a synonymous variant (p.Lys1192=), which, located in the last base of exon 26, leads to exon skipping and inframe deletion (p.Ser1135_Lys1192del) [35,36]. Very rare in the general population (GnomAD, ƒ = 1.59 × 10^−6^), the c.3576G>A has been described in many unrelated Italian AT patients with founder effect [37].

#### 3.2.2. *RAD51C*

LP variants in the *RAD51C* gene were found in a woman with bilateral BC and in two women with OC. The c.904+5G>T splice site variant was found in a patient with OC diagnosed at 40 years (P113). She referred OC also in her two paternal cousins, while her father and her mother had non-Hodgkin lymphoma and thyroid cancer, respectively (Figure S1b). The c.904+5G>T, described in BC and OC patients and families and in 0.002% of the general population (ExAC source), involves a consensus splice site of intron 6. Functional studies indicate that this variant causes aberrant mRNA splicing and the exon 6 skipping, leading to premature termination codon and a consequent truncated protein, p.(Val280Glyfs*11) [38].

The c.934C>T variant was identified in a bilateral metachronous BC case (P33), who carried also an *MSH2* VUS (c.1847C>G, p.(Pro616Arg). The lobular carcinoma at the left side was diagnosed at 35 years of age, whereas the high-grade triple-negative invasive DC (Ductal Carcinoma) occurred after 11 years. The father presented a BC at 54 years and, more recently, a PrC; a paternal uncle had three primary malignancies (cerebral, prostatic, and bladder cancers), whereas the mother and the grandmother had a CC and a BC, respectively (Figure S1a). The mutation analysis, extended to the parents, confirmed the maternal origin of both variants. The c.934C>T, p.(Arg312Trp), a non-conservative change at the ATPase domain of RAD51C described in OC cases, has been demonstrated to impair protein function [39]. In functional studies, indeed, the presence of this amino acidic substitution has been related to higher levels of DNA damage, altered RAD51 foci formation upon irradiation, and increased chromosomal instability [40].

The c.1026+5_1026+7del deletion was detected in a high serious grade (HSG) OC case (P110) with onset at 51 years. She referred a marked maternal familiarity for cancer, including OC, PrC, esophageal, and gastric cancers in first and second-degree relatives and leukemia and an early-onset brain tumor in cousins (Figure 2c). This rare variant (ƒ = 1.19 × 10^−6^, gnomAD data) has been reported in individuals with breast, ovarian, and uterine cancer. Functional studies have demonstrated that c.1026+5_1026+7del affects a consensus splice site in intron 8 of the *RAD51C*, leading to the exon 8 skipping and resulting in a frameshift mutation that causes a premature termination codon, p.(Arg322Serfs*22) [41,42,43].

### 3.3. MMR Genes Variants and Related Phenotypes

Variants in MMR genes were identified in two cases with BC. Both women had a high-grade hormone-responsive invasive DC, with onset at 39 and 56 years, respectively, and positive family history for malignancies among their relatives.

#### 3.3.1. *MLH1*

P67 patient carrying c.1696T>C in the *MLH1* gene reported BC in her mother and PrC in the two maternal uncles (Figure S1e). This rare variant (ƒ = 3.98 × 10^−6^, gnomAD data), replacing a tyrosine with histidine at codon 566 in the region interacting with EXO1 of the MLH1 protein p.(Tyr566His), is classified as LP by ACMG criteria, reported as VUS in ClinVar, and is still not present in the Insight database (https://www.insight-group.org/variants/databases/).

#### 3.3.2. *MSH2*

Proband P53 with the c.182A>C, p.(Gln61Pro) variant in *MSH2* gene reported cancer familiarity in both parental branches: PrC in the father and in the two paternal uncles, a not specified neoplastic disease in the third paternal aunt and in her daughter; BC in her mother, uterine cancer in the maternal grandmother, and PrC in the maternal uncle (Figure S1d). This variant, although reported with conflicting interpretation in Insight (VUS vs. P), is classified as LP according to ACMG criteria and has been found in a Lynch Syndrome case with OC and CC [44].

### 3.4. Cell Cycle Control Gene Variants and Related Phenotypes

#### *CHEK2* 

The c.470T>C variant was identified in two unrelated patients with unilateral hormone-responsive DC—invasive and moderately differentiated in the one case (P18) and in situ and well-differentiated in the remaining (P46) (Figure S2b). P18 patient also presented other primitive malignancies, including CC at 45 years, OC after 2 years, and GC at the age of 48. In addition, herather had multiple cancers: after a rectal carcinoma, a PrC occurred more recently. Similarly, P46 had a familiarity with rectal carcinoma (maternal uncle) but referred also OC in her younger sister and uterine adenocarcinoma in her mother (Figure 2a). In the same patient, the c.145G>A, p.(Ala49Thr) VUS in the *PMS2* gene was found. The *CHEK2* c.470T>C, p.(Ile157Thr) variant, located in the Forkhead-associated (FHA) domain of the encoded protein, is known to impair the CHEK2 binding to checkpoint proteins, including CDC25A, in response to DNA damage [45]. It also compromises the dimerization of the protein in a dominant-negative manner and alters the auto-phosphorylation [46], but does not affect the kinase activity of the protein [47]. In several studies, this variant has been associated with an increased risk of breast cancer, particularly lobular carcinoma [48], and additional tumors, including renal, prostatic, thyroid, and gastric cancer [49,50,51]. Based on co-segregation studies, the penetrance was complete in some families and incomplete in others [52].

The c.793-1G>A splicing variant was detected in a 46-year-old woman (P68) with papillary thyroid cancer at the age of 27 and first-degree familiarity for BC. She referred osteosarcoma in the son of her unaffected brother, a metachronous bilateral BC in her deceased mother, a PrC and a GC in the maternal uncle and grandfather, respectively. PrC was also present in three paternal uncles, while a fourth paternal uncle deceased with lung cancer. Segregation studies were performed in the brother but not in his young son, who denied his consent, despite his previous diagnosis of osteosarcoma and the detection of the *CHEK2* LP variant in his father (Figure S2g). The c.793-1G >A variant occurs in the splicing acceptor site of intron 6, causing an alternative splicing site downstream and a frameshift mutation, which leads to a premature termination codon and a consequent truncated protein p.(Asp265Thrfs*10) [53]. This variant has been found in individuals with BC, PrC, and in patients who underwent genetic testing for the high risk of hereditary cancer [54,55,56,57].

The c.1100del variant was detected in two women (P111 and P112) with bilateral metachronous hormone-responsive DC. Both cases had a post-menopausal onset and positive family history for BC in first and/or second-degree maternal relatives. One of them had also a diffuse mesenteric leiomiomatosis (Figure S2c,e). The c.1100del variant causes a frameshift and premature termination codon, p.(Thr367Metfs*15), responsible for the loss of the response to DNA damage and for the impairment of the CHEK2 kinase activity [47,52]. This variant has been reported in Li-Fraumeni Syndrome and also in several cancer types as breast, ovarian, prostatic, colon, thyroid malignancies [50,58,59,60].

The patient (P12) carrying the c.1136C>G, p.(Ser379Cys), variant had a foot melanoma at 49 years. Her mother had a GC at 45 years, her maternal uncle deceased at 60 years for a PC occurred two years earlier, and her maternal aunt, as well as her grandmother, had a BC. Moreover, her father had head-neck and lung cancer, one of his three sisters a BC, and his brother a lung cancer (Figure S2d). Molecular analysis of this proband identified also a VUS in *RAD50* (c.1452+7T>G). The c.1136C>G change replaces a highly conserved serine with cysteine at codon 379 of the CHEK2 protein, p.(Ser379Cys). It is located in exon 11 of the *CHEK2* gene, a hot-spot region of 61 base pairs length, where all identified variants are classified as pathogenic (Varsome source). Prediction tools (Mutation Taster, SIFT, Provean) consider this variant as damaging, and, based on ACMG criteria, it can be classified as LP.

The c.1169A>C variant was found in a patient (P04) with a Mullerian sarcoma at the age of 47 and BC in her relatives. Her mother, her sister, and a maternal cousin indeed had BC at 47, 57, and 23 years, respectively. Two maternal uncles had also malignancies—one pulmonary and the other colorectal. However, colon cancer familiarity was stronger among paternal relatives, occurring in her father, uncle, and grandfather (Figure S2a). The c.1169A>C change replaces a highly conserved tyrosine with serine at codon 390 of the kinase domain of the protein, p.(Tyr390Ser) [52,61]. In functional studies, p.(Tyr390Ser) CHEK2 protein has not exhibited any kinase activity [62]. This variant has been found in individuals with a personal and/or family history of breast and/or ovarian cancer [63,64] and, rarely, in the general population (ƒ = 2.4 × 10^−5^, gnomAD data).

The c.1367C>T variant in the *CHEK2* gene was detected in a male proband (P60) with an in situ cribriform carcinoma at the age of 71 years and familiarity, both maternal and paternal, for BC and OC (Figure S2f). The c.1367C>T change replaces serine with leucine at codon 456 of the CHEK2 protein, p (Ser456Leu). It is located in exon 13, a region where the 90% of identified variants are pathogenic (Varsome source). Not reported in the gnomAD population database, it is predicted to be deleterious by computer-based algorithms used in Varsome (https://varsome.com/). Despite reported only one time in ClinVar as VUS, it can be classified as LP based on ACMG criteria.

### 3.5. DNA Repair Helicase Gene Variant-Related Phenotypes

#### *RECQL* 

The new *RECQL* variant c.363_364del was identified in a 72-year-old male proband (P90) with BC at 59 years and PrC at 70, having a sister deceased at 56 years for pulmonary cancer and BC at 38 years (Figure 2d). This small deletion in *RECQL* was also excluded in the healthy 49 years old daughter of the proband. This variant is located in the helicase Rec-A like domain A1 (amino acid residues 63–281) of RECQL protein, containing the highly conserved signature helicase motifs of the SF-2 superfamily. This variant is predicted to cause a premature termination codon with consequent production of a truncated protein, p.(Cys122Leufs*43), and loss of the helicase activity. Germline mutations of *RECQL* were identified in patients with hereditary BC. In independent studies, damaging mutations in *RECQL* have been related to increased breast cancer risk and to genomic instability [19,29,65].

### 3.6. VUS Variants

Twenty-three cases had exclusively VUS. All but one carried a single variant. The P103 patient, carrier of two VUS (c.4703A>G, p.(His1568Arg), in *ATM* and c.7667C>T, p.(Ser2556Leu), in *APC*), had a low-grade hormone-responsive BC and a family history for BC and other cancers. In two unrelated women, the same VUS in the *PALB2* gene (c.3436C>A) was identified, one with an early onset BC and one with OC. The remaining probands were carriers of single VUS. Four probands were the carrier of VUS in the *ATM* gene: three women with BC and one with OC. All but the bilateral BC woman had first and/or second-degree relatives with BC (4), PrC (1), PC (1), and other cancers (4). The two cases with VUS in *CHEK2* had premenopausal familial BC and early-onset BC (38 years), melanoma, and positive family history for OC and uterine cancer, respectively. *BRIP1* was found mutated in two cases: one woman had OC and relatives with BC, while the other one had BC at 42 years of age and positive family history for early-onset BC, PrC, brain, and colorectal cancer. The remaining 11 patients had VUS in 11 different genes. The two women with early onset of BC (at 36 and 38 years, respectively) had variants in *CDH1* and *PMS2*. The patient with multiple cancers (breast and renal) had a VUS in the *EPCAM* gene. The male case of low-grade hormone-responsive BC, with onset at 43 years, was a *MUTYH* VUS heterozygous carrier. The other women had BC and a positive family history of many types of cancers. The correlation genotype-phenotype of patients with VUS are summarized in Table S3.

### 3.7. Genotype-Phenotype Correlations

For each subgroup of the cohort (female BC, male BC, OC, other cancers, and/or positive familiarity), clinical features were first compared between LP/P variants carriers (P/LP patients) and probands with not informative tests (NI, that is patients with VUS, B/LB, or no variants), then between P/LP patients and each of the two NI subclasses: negative (N, that is patients with B/LB or no variants) and VUS, and finally, between the latter two (Tables S4–S7)

Compared to NI-fBC and its subclasses, in P/LP-fBC patients, a bilateral disease was significantly more frequent. Hormone responsive and Her2 (Human epidermal growth factor receptor 2) positive phenotype was rarer in P/LP BC cases and significantly higher in NI ones. In addition, familiarity for BC was less recurrent among relatives of P/LP women compared to NI, N, and VUS cases, but splitting up first and second-degree kinds, this difference remained significant only in the latter. Conversely, although not statistically significant, a trend of higher PrC familiarity emerged among P/LP-fBC first-degree relatives. No other differences were found in other demographic, clinical, and familial features (Table S4). Among OC patients, P/LP OC was younger than NI, N, and VUS OC cases, and, in their families, BC and PrC recurred more frequently in the first degree and second-degree relatives, respectively (Table S5). No differences emerged comparing mBC with and without P/LP variants (Table S6), while, among cases with other cancers and/or malignancies in relatives, P/LP variants were detected exclusively in affected probands rather than in healthy ones (Table S7).

Comparison among PC patients could not be performed for the small number of probands with this diagnosis (three cases).

## 4. Discussion

The present study aimed at detecting pathogenic variants related to hereditary cancers by multigene panel testing. In total, 113 consecutive individuals with personal or family history of breast, ovarian, or pancreatic cancer and without P/LP variants in *BRCA1* and *BRCA2* genes were analyzed. By applying NGS technologies in our cohort, we investigated the frequency of germline deleterious variants in *APC*, *ATM*, *BARD1*, *BRIP1*, *CDH1*, *CDK4*, *CDKN2A*, *CHEK2*, *EPCAM*, *MLH1*, *MRE11*, *MSH2*, *MSH6*, *MUTYH*, *NBN*, *PALB2*, *PMS2*, *PTEN*, *RAD50*, *RAD51C*, *RAD51D*, *RECQL1*, *SMAD4*, *STK11*, and *TP53* genes, all involved in DDR system. The choice of including these genes in our panel was based on several considerations.

The DNA damage response plays a critical role in maintaining genomic stability, and hereditary mutations in DDR genes often confer cancer susceptibility. Bi-allelic mutations in DSBR genes and in other DDR genes are indeed the basis of cancer-prone recessive hereditary syndromes, such as Ataxia–Telangiectasia, Nijmegen Breakage Syndrome, and Fanconi Anemia. Moreover, alterations in the MMR genes result in Lynch Syndrome, leading to an increased incidence of gastrointestinal, endometrial, and ovarian cancers.

Most chemotherapy agents currently used in cancer therapy cause DNA damage. DDR pathway inhibitors are now used to make cancer cells more sensitive to chemotherapy—an approach called synthetic lethality. In the era of PARP inhibitors, employed in *BRCA* carriers with OC and metastatic BC, the identification of further potential targets in other HRR (Homologous Recombination Repair) genes could provide new therapeutic opportunities for this and other cancers related to defects in DDR genes.

Genetic testing for hereditary cancer predisposition has evolved rapidly. Many genes included in multigene panels have inaccurate estimations about the degree of associated cancer risk, and there is no consensus on when to test a particular gene or how to manage an identified P/LP variant. A survey conducted in 61 centers from 20 countries by the clinical group of ENIGMA (Evidence-Based Network for the Interpretation of Germline Mutant Alleles) in 2018 [66] showed that, beyond *BRCA1/2*, only a small number of genes are currently analyzed worldwide, and management guidelines are limited. On the other hand, the clinical utility of detecting pathogenic germline variants in high and moderate penetrance genes is strong, as recommended by NCCN guidelines for cancer prevention, surveillance, and management.

In total, 14 different P/LP variants in 6/25 DDR pathway genes were identified in 16 probands. Despite the rarity of each damaging mutation, the overall pathogenic variants rate in these 25 DDR genes was 14% (16/113 unrelated *BRCA1/2*-negative cases). Moreover, 23 probands carried VUS (20%), whereas 74 (66%) were negative (Figure 1a). In several NGS studies of patients with BC and/or OC, beyond *BRCA1* and *BRCA2*, *CHEK2* has been one of the most frequently mutated genes [67,68,69]. This data was confirmed in our sample, in which 7.1% (8/113) of analyzed patients were found carriers of deleterious mutations in the *CHEK2* gene. *RAD51C*, with a frequency of 2.6% (3/113), was the other most mutated gene in our patients. These data supported the rationale of including this gene in the NGS panels for the assessment of BC/OC risk.

Germline *CHEK2* P/LP variants have been associated with Li-Fraumeni like syndrome, BC, and other cancers, including prostatic, gastrointestinal, and, although still debated, OC [70]. In our study, P/LP variants in *CHEK2* were detected in women with monolateral and bilateral BC, who referred at least one or more different types of malignancies, including BC, OC, and gastrointestinal cancers, in their relatives. Moreover, the c.793-1G>A variant was identified in a woman with a family history of osteosarcoma and young-onset BC that suggested a Li-Fraumeni like syndrome. Intriguingly, the proband had papillary thyroid cancer, recently described in *CHEK2* patients [59]. Unfortunately, segregation studies could not be performed in the deceased parents, and we could not determine if the variant, detected also in her asymptomatic brother, was inherited from the maternal branch, in which breast, gastrointestinal, and brain cancers occurred, or from the paternal side, where three paternal uncles had PrC.

*CHEK2* LP variants, c.1136C>G, p.(Ser379Cys) and c.1169A>C, p.(Tyr390Ser), were detected in a woman with melanoma (*P12*) and in a Mullerian sarcoma case (*P04*), respectively, both referring BC and gastrointestinal cancers in relatives. Previous studies have demonstrated that *CHEK2* c.1100del heterozygotes have a two-fold risk of malignant melanoma compared to non-carriers, while no clear associations have emerged between Mullerian sarcomas or, more generally, isolated uterine tumors and *CHEK2* [71,72]. Nevertheless, the recurrence of BC and GC and CC in many relatives of these two families was in line with the typical phenotype related to this gene.

Recently *CHEK2* pathogenic variants have been considered a proven genetic risk factor for male BC. Although many authors have confirmed this association worldwide [70], the detection of P/LP *CHEK2* variants in Italian male BC cases was poor [73]. Despite this data, among our nine mBC cases, one of the three mutated patients, who referred BC and OC among paternal and paternal relatives, carried the c.1367C>T LP variant in *CHEK2*.

Finally, the c.470T>C, p.(Ile157Thr) *CHEK2* variant, described in BC and many other types of malignancies, was found in a proband with four primary different tumors (BC, OC, CC, and GC), who referred multiple cancers also in her father (RC and PrC). *CHEK2* damaging variants have been found in families with multiple primary cancers [72,74,75]. However, to our knowledge, a high number of primary tumors has never been described in patients carrying the *CHEK2* c.470T>C mutation. Unfortunately, parental segregation studies could not be performed, preventing the investigation of the variant in her affected father.

Mutations in the *RAD51C* gene, encoding a protein involved in HR, were found in 3/113 cases analyzed—two patients with OC and a woman with bilateral BC. Deleterious variants in this gene have been associated with a higher risk of epithelial ovarian carcinoma, especially with early onset [76]. Although a well-defined genetic risk factor for OC, the role of *RAD51C* in BC, is still debated [38,77]. The woman carrying the *RAD51C* missense gene alteration had a bilateral metachronous BC, a lobular carcinoma on the left, and a high-grade triple-negative invasive DC occurred 11 years later on the right. Intriguingly, in patients with BC and damaging mutations in *RAD51C*, triple-negative cancers recurred many times [78,79].

Bi-allelic P/LP variants in the *ATM* gene cause Ataxia–Telangiectasia (AT), a neurodegenerative progressive disease complicated by immunodeficiency and cancer predisposition. Germline *ATM* heterozygous carriers are about 0.75–1% of the population. Mono-allelic variants of this gene are proven as moderate risk factors for malignancies, including breast, pancreatic, prostatic, and other solid cancers. In our cohort, *ATM* pathogenic variants were identified in two probands with multiple cancers. The *P29* patient, affected by BC and melanoma, had a paternal family history of different types of malignancies previously described in *ATM* heterozygous patients; the *P89* patient had a metastatic PC and a previous diagnosis of GC. In his family, nine relatives deceased for gastric cancer. Although not frequently, pathogenic variants in *ATM* have been previously described in patients with GC [80,81,82]. PC remains one of the most lethal solid malignancies. The identification of damaging mutations in DDR system genes, including *ATM*, in 17–25% of this type of cancer and the recent suggestion that PARP inhibitors could have therapeutic potential in cancers with loss or mutation of *ATM* are opening up the possibility of new therapies, such as platinum and more recently PARP inhibitors, also in *ATM*-mutated patients with PC [83,84,85].

LP variants in the MMR genes *MLH1* and *MSH2* were found in two women with BC. Although BC is not included in the spectrum of Lynch Syndrome (LS)-related malignancies, an association between LS germline mutations and this cancer has been recently suggested. Many studies indeed have reported a higher risk of BC in patients with LS and a higher frequency of MMR gene variants in BC cases [21,86]. However, women with pathogenic variants in MMR genes are not usually advised to increase breast cancer screening. Furthermore, immunohistochemistry for MMR proteins is not performed for the identification of deficient breast cancers. Studies able to clarify this association are needed also for therapeutic implications. Checkpoint inhibitors, such as pembrolizumab, are becoming available treatments for all microsatellite instable-high and/or MMR deficient solid tumors (including breast cancer) [87].

In this study, we described a new frameshift deletion c.363_364del in the *RECQL* gene in a male with infiltrating ductal breast and prostatic cancer at 59 and 70 years of age, respectively, with a positive family history for breast and lung cancer (Figure 2d). *RECQL* encodes a protein that is part of a family of five RECQ helicases, including at least three implicated in cancer-prone syndromes, such as Bloom Syndrome, Werner Syndrome, and Rothmund-Thomson Syndrome. These diseases are indeed caused by bi-allelic mutations in the *BLM*, *WRN*, and *RECQ4* genes, respectively [88]. *RECQL* is a helicase involved not only in the unwinding of the DNA but also in the promotion of complementary single-strand DNA annealing. Its role in DDR has been clearly proven by many studies that have demonstrated chromosomal instability, stalled and collapsed replication forks, oxidative damage, higher DSBs in cells with a deficit of RECQL [29]. In 2015, two independent research groups associated the damaging variants in *RECQL* with a higher risk of BC in studies conducted in Polish and Canadian populations [65,89]. However, this correlation was not confirmed in subsequent studies [90]. More recently, mutations in *RECQL* emerged as a moderate risk factor for BC in a cohort of African American women, indicating that mutations in the *RECQL* gene confer a moderate risk of BC [91]. These conflicting correlations could be explained by penetrance variability due to the consequences of the different types of the identified variants on the protein function and to the investigated cohorts belonging to different ethnicities [92].

More than 20% of patients have carried VUS. This type of inconclusive result is a hard challenge to face. With the advent of panel analysis, the number of VUS has increased exponentially, and still, too often, their interpretation remains tangled and blurred. Unfortunately, in most cases, VUS causes difficulty in risk assessment, sometimes overtreatment, and usually anxiety in carriers. Bioinformatic analysis, functional studies, and periodical updates performed by international consortia [93] are the currently available strategies to shed light on this issue [94].

Laboratories and clinicians should collaborate in order to guarantee periodical re-evaluations and updates on their variants to VUS carriers [27].

The comparison of clinical and familial features between patients with and without P/LP variants brought out interesting suggestions. In our cohort, P/LP variants were more frequent in women with bilateral BC, as reported before [95]. There was not a prevalent histological phenotype in mutated BC women compared to BC cases without damaging mutation nor a strong recurrence of specific types of cancers among relatives. Intriguingly, but not surprisingly, a trend of higher frequency of prostatic cancer among relatives of BC women with P/LP variants emerged. This association was stronger in relatives of mutated OC patients. Pathogenic variants in DDR and MMR genes, usually analyzed in suspected hereditary BC and OC, have been recently associated also with hereditary prostatic cancer [96].

A wider number of probands and the availability of segregation among relatives would have given the opportunity to furtherly strengthen the association between the identified variants and the predisposition to cancer. Future studies investigating P/LP variants in DDR genes in many BC, OC, and PC cases, familial or sporadic, and in their relatives would be necessary to better define the role and the weight of these genes in determining malignancies.

In conclusion, in this study, we described that 14% of non-*BRCA* patients with BC/OC tumors are carriers of pathogenic variants in other genes, particularly *CHEK2*, *RAD51C*, *ATM*, *MLH1*, *MSH2*, *RECQL*, all related with the *BRCA1/2* DNA repair pathway. This result shows that the DDR genes panel significantly increases the diagnostic power in patients with personal and/or family history of breast/ovarian and pancreatic cancers.

The identification of mutations in genes involved in DNA damage response, other than *BRCA*, explains the strong tumor recurrence in some families and may contribute to the development of new and more specific clinical management programs and pave the way to new therapeutic opportunities.

## Figures and Tables

**Table 1:** DNA damage repair (DDR) genes analyzed in this study.

Panel Gene	Syndrome	Main Pathway	Cancer Related	Reference
*ATM*	Ataxia Telangiectasia (AR)	DOUBLE STRAND BREAKS REPAIR (Homologous Recombination)	Breast, ovarian, pancreatic	[2,24,26]
*PALB2*	Fanconi Anemia (AR)			[24,26,27]
*MRE11A*	Ataxia Telangiectasia-like disorder (AR)		Breast	[4,27]
*RAD50*	Nijmegen breakage syndrome-like disorder (AR)			[4,28]
*BARD1*				[6]
*NBN*	Nijmegen Breakage Syndrome (AR)		Breast, ovarian	[26]
*BRIP1*	Fanconi Anemia (AR)			[7]
*RAD51C*				[27]
*RAD51D*				[27]
*STK11*	Peutz-Jeghers Syndrome (AD)		Colorectal, breast, pancreatic, gastric, small intestine, cervical, ovarian	[2,11]
*MSH2*	Lynch Syndrome (AD)	MISMATCH REPAIR	Colorectal, endometrial, ovarian, gastric, urothelial, pancreaticobiliary, cutaneous sebaceous neo-plasms, brain	
*MLH1*				
*MSH6*				[2,26]
*PMS2*				
*EPCAM*				
*MUTYH*	MYH-Associated polyposis (AR)	BASE EXCISION REPAIR	Colorectal, duodenal, breast	[22]
*RECQL*		DNA REPAIR (helicase)	breast	[29]
*TP53*	Li-Fraumeni Syndrome (AD)	cell cycle control	Breast, sarcoma, brain, adrenocortical, leukemia, gastric	[2,26,27]
*PTEN*	Cowden Syndrome (AD)		Colorectal, breast, endometrial, thyroid, renal	[2,16,26,27]
*CHEK2*	Li-Fraumeni variant (AD)		Breast ovarian	[2,5,26]
*CDH1*	Hereditary diffuse gastric cancer (AD)		Gastric, breast	[2,26,27]
*CDK4*	Familial melanoma (AD)		Melanoma	[30]
*CDKN2A*			Melanoma, pancreatic	[2]
*SMAD4*	Juvenile polyposis Syndrome (AD)		Colorectal, Gastric	[17]
*APC*	Familial adenomatous polyposis (AD)		Colorectal, small intestine, ampullary, gastric, desmoid, thyroid	[13]

List of the 25 genes selected for this study. AD: autosomal dominant; AR: autosomal recessive.

**Table 2:** Main clinical variables of the study sample.

	*n* pts (F/M)	Mean Age Tot ± SD (Min–Max)	Mean Age at Onset Tot ± SD (Min–Max)	*n* Pts Onset ≤ 40 Years	Family History 1st, 2nd Degree (BC, OC, PrC, PC other C, multiple C)
BC	87 (87/0)	54.39 ± 10.28 (36–83)	49.25 ± 10.35 (30–82)	16	82 (68, 18, 22, 9, 59, 14)
Male BC	7 (0/7)	63.1 ± 11.8 (49–78)	60.7 ± 13.2 (43–76)	0	6 (4, 2, 1, 0, 6, 3)
OC	9 (9/0)	59.56 ± 8.75 (46–71)	52.33 ± 14.04 (28–68)	2	9 (6, 3, 1, 0, 7, 0)
PC	3 (1/2)	67.7 ± 2.1 (65–69)	65.3 ± 0.6 (65–66)	0	3 (1, 0, 1, 0, 3, 0)
Fam	7 (7/0)	54.14 ± 8.63 (45–67)	-	-	7 (5, 2, 3, 3, 6, 3)
Total	113 (104/9)	55.65 ± 10.37 (36–83)	50.24 ± 11.19 (27–82)	19	107 (84, 25, 28, 12, 81, 20)

BC, breast cancer; OC, ovarian cancer; PC, pancreatic cancer; PrC, prostatic cancer; C, cancer; pts, patients; F, female; M, male; Fam, familiarity.

**Table 3:** P/LP and VUS gene variants detected in the sample.

Gene	Locus	Transcript	dbsnp	cDNA (HGVS)	Protein	Type of Variants	Gnomad	ACMG Classification	Count
*APC*	chr5:112176574	NM_000038.5	rs933729249	c.5283C>G	p.(Asn1761Lys)	missense	/	VUS	1
	chr5:112176656		rs1554086666	c.5365G>C	p.(Val1789Leu)	missense	/	VUS	1
	chr5:112178958		rs761133356	c.7667C>T	p.(Ser2556Leu)	missense	0.000012	VUS	1
*ATM*	chr11:108114838	NM_000051.3	rs771685059	c.655T>C	p.(Cys219Arg)	missense	0.00000796	VUS	1
	chr11:108115727		rs747727055	c.875C>T	p.(Pro292Leu)	missense	0.00000806	LP	1
	chr11:108151895		rs587776551	c.3576G>A	p.(Lys1192=)	synonymous	0.0000159	P	1
	chr11:108163473		rs1064795495	c.4564G>A	p.(Gly1522Ser)	missense	0.00000398	VUS	1
	chr11:108164131		rs368830730	c.4703A>G	p.(His1568Arg)	missense	0.0000398	VUS	1
	chr11:108181006		rs56399311	c.5882A>G	p.(Tyr1961Cys)	missense	0.0000398	VUS	1
	chr11:108198368		/	c.6976-4A>G	p.?	intronic	/	VUS	1
	chr11:108205751		rs759779781	c.8066A>G	p.(Glu2689Gly)	missense	0.00000398	VUS	1
*BARD1*	chr2:215661789	NM_000465.3	rs1060501308	c.211T>A	p.(Cys71Ser)	missense	/	VUS	1
*BRIP1*	chr17:59937216	NM_032043.2	/	c.146G>A	p.(Gly49Glu)	missense	/	VUS	1
	chr17:59934442		rs889877039	c.356A>G	p.(Asn119Ser)	missense	0.000137	VUS	1
*CDH1*	chr16:68842738	NM_004360.4	rs786203207	c.674T>C	p.(Ile225Thr)	missense	/	VUS	1
*CDKN2A*	chr9:21970943	NM_001195132.1	rs587781733	c.415G>A	p.(Gly139Ser)	missense	/	VUS	1
*CHEK2*	chr22:29121228	NM_007194.3	rs587781279	c.444+3A>G	p.?	intronic	/	VUS	1
	chr22:29121087		rs17879961	c.470T>C	p.(Ile157Thr)	missense	0.001	LP	2
	chr22:29107974		rs121908702	c.715G>A	p.(Glu239Lys)	missense	/	VUS	1
	chr22:29106048		rs730881687	c.793-1G>A	p.(Asp265Thrfs*10)	splicing	/	P	1
	chr22:29091857		rs555607708	c.1100delC	p.(Thr367Metfs*15)	frameshift	0.002	P	*2*
	chr22:29091788		rs200928781	c.1169A>C	p.(Tyr390Ser)	missense	/	LP	1
	chr22:29091821		rs267606211	c.1136C>G	p.(Ser379Cys)	missense	/	LP	1
	chr22:29091123		rs876659827	c.1367C>T	p.(Ser456Leu)	missense	/	LP	1
*EPCAM*	chr2:47602397	NM_002354.2	rs864622724	c.450C>G	p.(His150Gln)	missense	/	VUS	1
*MLH1*	chr3:37083787	NM_000249.3	rs730881743	c.1696T>C	p.(Tyr566His)	missense	/	LP	1
*MSH2*	chr2:47630512	NM_000251.2	rs587779113	c.182A>C	p.(Gln61Pro)	missense	/	LP	1
	chr2:47702191		rs41295288	c.1787A>G	p.(Asn596Ser)	missense	0.000318	VUS	1
	chr2:47702251		rs587779965	c.1847C>G	p.(Pro616Arg)	missense	/	VUS	1
*MSH6*	chr2:48027323	NM_000179.2	rs1060502883	c.2201T>A	p.(Val734Glu)	missense	/	VUS	1
*MUTYH*	chr1:45798518	NM_001128425.1	rs890418965	c.505-12T>G	p.?	intronic	/	VUS	1
*PALB2*	chr16:23614905	NM_024675.3	rs879254033	c.3436C>A	p.(Gln1146Lys)	missense	/	VUS	2
*PMS2*	chr7:6045541	NM_000535.6	rs1583418527	c.145G>A	p.(Ala49Thr)	missense	/	VUS	1
	chr7:6027143		rs587782640	c.1253C>T	p.(Ser418Phe)	missense	/	VUS	1
	chr7:6022480		rs201671325	c.2149G>A	p.(Val717Met)	missense	0	VUS	1
*RAD50*	chr5:131925536	NM_005732.3	/	c.1452+7T>G	p.?	intronic	/	VUS	1
	chr5:131939174		/	c.2390G>A	p.(Arg797Lys)	missense	/	VUS	1
*RAD51C*	chr17:56798178	NM_058216.2	rs587782702	c.904+5G>T	p.(Val280Glyfs*11)	intronic	/	LP	1
	chr17:56801430		rs730881932	c.934C>T	p.(Arg312Trp)	missense	0.00001	LP	1
	chr17:56809908		rs587781410	c.1026+5_1026+7del	p.(Arg322Serfs*22)	intronic	/	LP	1
*RECQL*	chr12:21643163	NM_032941.2	New	c.363_364del	p.(Cys122Leufs*43)	frameshift	/	P	1
	chr12:21643150		rs1267616869	c.377C>T	p.(Pro126Leu)	missense	/	VUS	1
*STK11*	chr19:1219382	NM_000455.4	rs369764220	c.434A>G	p.(Glu145Gly)	missense	0.0000134	VUS	1

Pathogenic, likely pathogenic, and VUS variants detected by 25 genes of cancer panel among 113 patients with a history familial/personal of cancer. Abbreviations: dbSNP, Single Nucleotide Polymorphism Database (https://www.ncbi.nlm.nih.gov/snp/); rs, reference SNP; HGVS: Human Genome Variation Society (http://www.HGVS.org/varnomen); GnomAD, Genome Aggregation Database (https://gnomad.broadinstitute.org/); ACMG, American College of Medical Genetics and Genomics; P, pathogenic; LP, likely pathogenic; VUS, variant of uncertain significance. Variants were annotated according to the current HGVS nomenclature; p.? consequence on protein structure unknown.

**Table 4:** Clinical characteristics of probands with P/PL variants.

ID Sample	Sex	Gene	P/LP Variants	Other Variants (VUS )	Age (Years)	Age at Onset (Years)	Tumor Site	Histological Diagnosis	Grading	Other Personal History of Cancer (Onset in Years)	Family History of Cancer (n of 1st and 2nd Degree Affected Relatives)
*P29*	F	*ATM*	c.875C>T p.(Pro292Leu)	RECQL c.377C>T p.(Pro126Leu)	44	43	breast (left)	DCIS	G1	M(42)	M (1); CNSC (1)
*P89*	M	*ATM*	c.3576G>A p.(Lys1192=)	-	66	66	pancreas	Adenocarcinoma	na	GC(45)	GC (8)
*P113*	F	*RAD51C*	c.904+5G>T p.(Val280Glyfs*11)	-	46	43	ovary	HSGC	na	-	NHL(1); OC (2), TC (1)
*P33*	F	*RAD51C*	c.934C>T p.(Arg312Trp)	c.1847C>G p.(Pro616Arg)	47	35	breast (left); breast (right)	CLI; IDC	G3	-	BC (1 + 1); CC (1); BlC (1), CNSC(1)
*P110*	F	*RAD51C*	c.1026+5_1026+7del p.(Arg322Serfs*22)	-	53	51	ovary	HSGC	na	-	OC (1), GC (1), EC (1); PrC (1); LC (1)
*P67*	F	*MLH1*	c.1696T>C p.(Tyr566His)	-	39	39	breast (right)	IDC	G3	-	BC (1); PrC (2)
*P53*	F	*MSH2*	c.182A>C p.(Gln61Pro)	-	58	56	breast (left)	IDC	G3	-	PrC (3 + 1), UC (1); unk C (1)
*P90*	M	*RECQL*	c.363_364del p.(Cys122Leufs*43)	-	72	59	breast (right)	IDC	G2	PrC	BC (1); LC (1)
*P18*	F	*CHEK2*	c.470T>C p.(Ile157Thr)	-	47	42	breast (left)	CDI	G2	CC, OC, GC	PrC (1); RC (1)
*P46*	F	*CHEK2*	c.470T>C p.(Ile157Thr)	PMS2 c.145G>A p.(Ala49Thr)	51	50	breast (right)	DCIS	G1	-	OC (1); UC (1); RC (1)
*P68*	F	*CHEK2*	c.793-1G>A p.(Asp265Thrfs*10)	-	47	27	tyroid	papillary	na	-	BC (1), PrC(1 + 3); GC (1); LC (1); S(1)
*P111*	F	*CHEK2*	c.1100del p.(Thr367Metfs*15)	-	56	50	breast (bil)	IDC	G2 (right) G1 (left)	-	BC (1)
*P112*	F	*CHEK2*	c.1100del p.(Thr367Metfs*15)	-	66	64	breast (bil)	IDC	G3 (right) G2 (left)	dML	BC (2 + 1), unk C (1)
*P12*	F	*CHEK2*	c.1136C>G p.(Ser379Cys)	RAD50 c.1452+7T>G	49	49	skin/foot	melanoma	na	-	BC (2 + 1); GC (1); PC (1); LC (2), head-neck C (1)
*P60*	M	*CHEK2*	c.1367C>T p.(Ser456Leu)	-	71	71	breast	DCIS	G2	-	BC (2 + 1), OC (1 + 1); LC (1)
*P04*	F	*CHEK2*	c.1169A>C p.(Tyr390Ser)	-	53	52	uterus	Mullerian sarcoma	na	-	BC (2), CC (3 + 1)

Abbreviations: DCIS, in situ ductal carcinoma; IDC, infiltrating ductal carcinoma; CLI, infiltrating lobular carcinoma; HGSC, high-grade serous carcinoma; BC, breast cancer; OC, ovarian cancer; PrC, prostate cancer; CNSC, central nervous system cancer; LC, lung cancer; M, Melanoma; GC, gastric cancer; NHL, non-Hodgkin lymphoma; RC, renal cancer; CC, Colon Cancer; EC, esophageal cancer; TC, thyroid cancer; PC, pancreatic cancer; S, sarcoma; BlC, bladder cancer; UC, uterine cancer; HNC, head-neck carcinoma; dML, diffuse mesenteric leyomiomatosis; unk C, cancer at a not specified site.

## Supplementary Materials

The following are available online at https://www.mdpi.com/2077-0383/9/9/3003/s1, Figure S1: Pedigrees of five families with P/LP variants, Figure S2: Pedigrees of CHEK2 families. Individuals with any cancer are shown as filled circles, Table S1: Design of multigene panel, Table S2: Sequencing metrics by NGS Analysis, Table S3: Clinical characteristics of probands with VUS variants, Table S4: Females with breast cancer (fBC), Table S5: Patients with ovarian cancer, Table S6: Males with breast cancer (mBC), Table S7: Probands enrolled for their positive family history.
