# Supplementary material for: Beyond BRCA1 and BRCA2: Deleterious Variants in DNA Repair Pathway Genes in Italian Families with Breast/Ovarian and Pancreatic Cancers
Source: J Clin Med. 2020 Sep 17;9(9):3003. doi: 10.3390/jcm9093003 (PMC7563793; doi:10.3390/jcm9093003)
Supplement: Supplementary file 1 [file jcm-09-03003-s001.pdf]

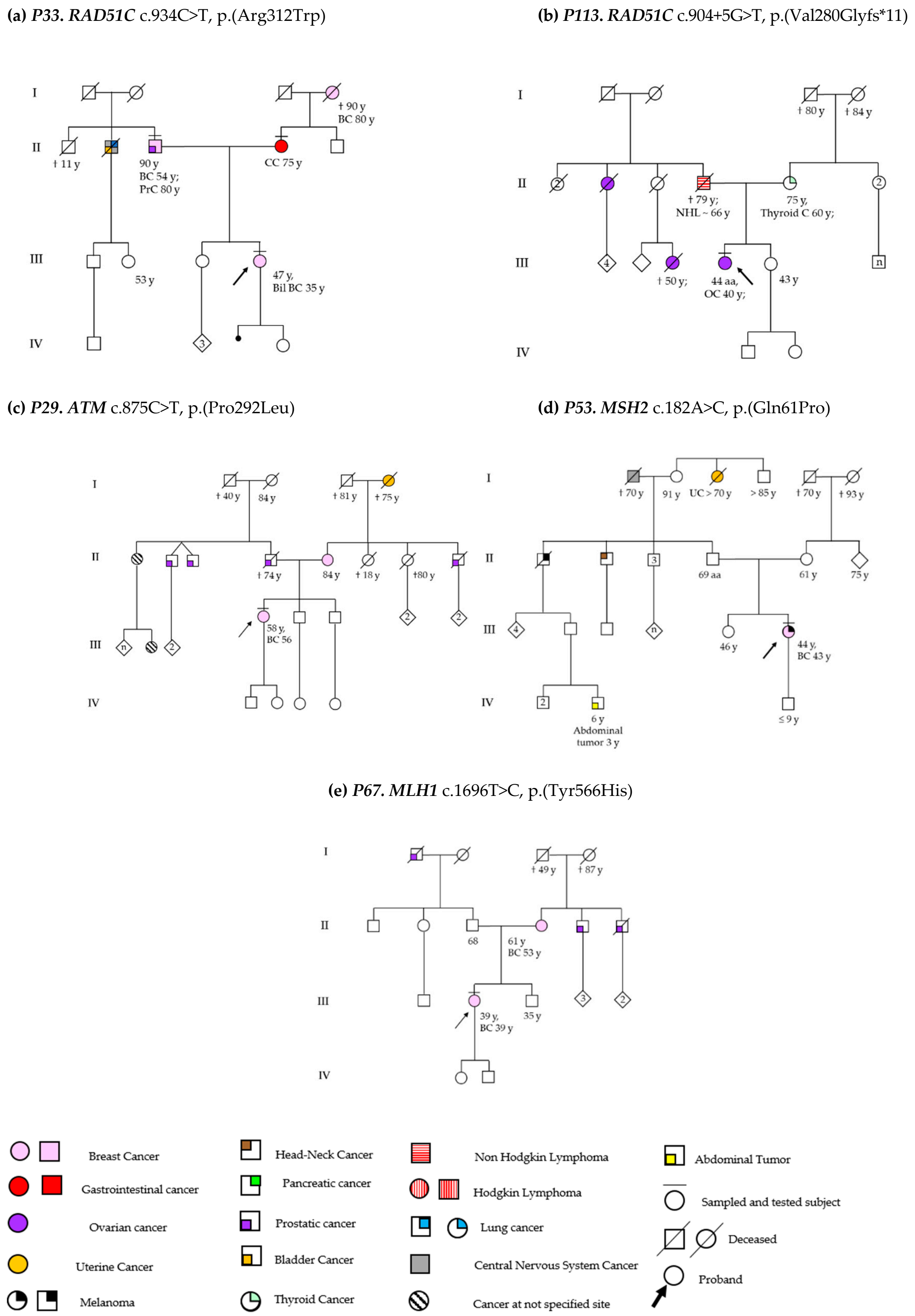

Figure S1. Pedigrees of five families with P/LP variants. Individuals with any cancer are shown as filled circles or square. Arrows show a proband with P/LP variant in **a**: (**a**, **b**) *RAD51C* families, (**c**) *ATM* family, (**d**) *MSH2* family, (**e**) *MLH1* family. The tested subject is indicated with a horizontal line above the circle or square. BC, breast cancer; OC, ovarian cancer CC, colon cancer; UC, uterine cancer; NHL, non-Hodgkin lymphoma; PrC, prostatic cancer; Bil BC, bilateral breast cancer.

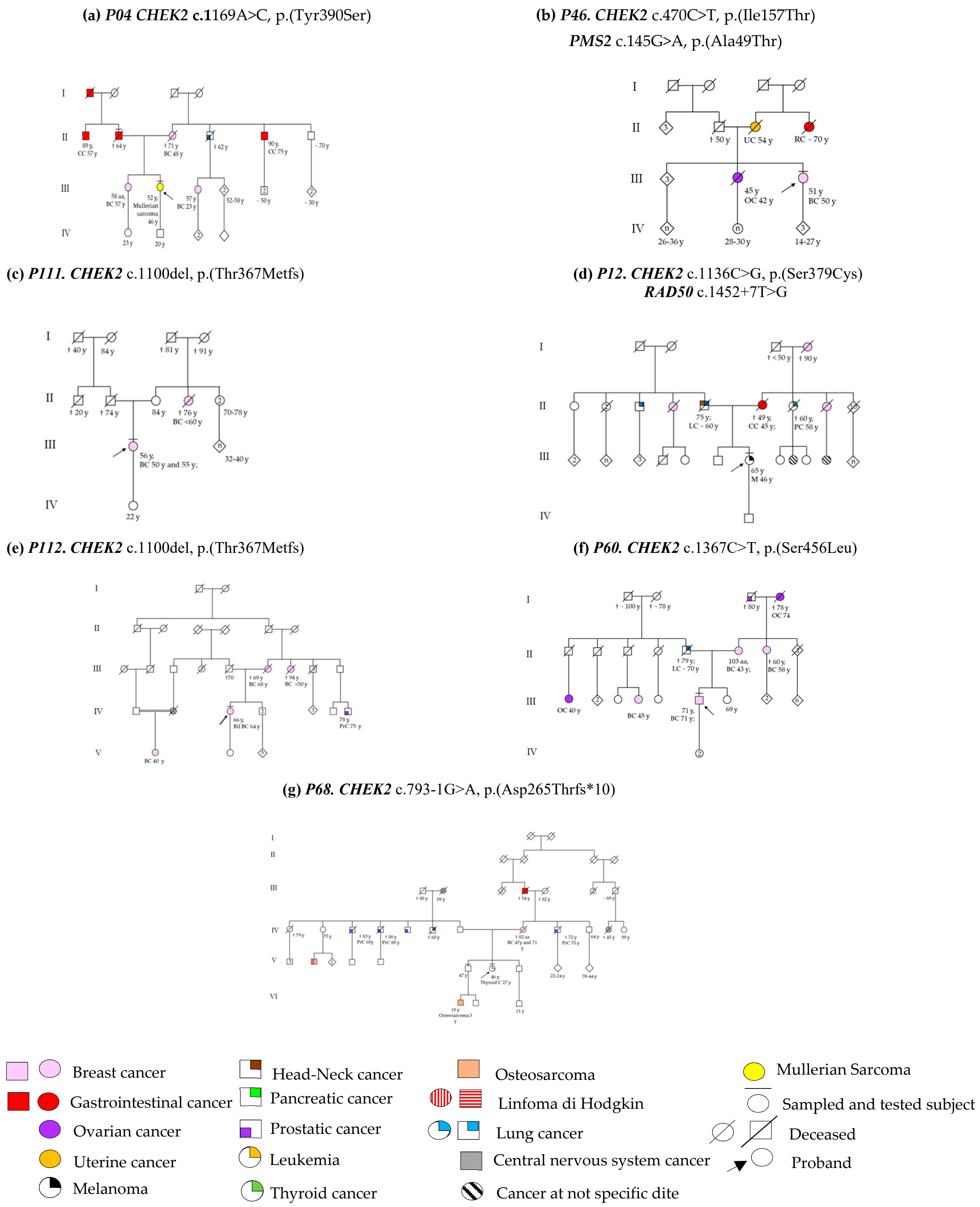

Figure S2. Pedigrees of *CHEK2* families. Individuals with any cancer are shown as filled circles. Arrows show a proband with P/LP variant. The tested subject is indicated with a horizontal line above the circle or square. BC, breast cancer; OC, ovarian cancer; CC, colon cancer; UC, uterine cancer; RC, rectal cancer; M, melanoma, LC, lung cancer; PC, pancreatic cancer; Bil BC, bilateral breast cancer; PrC, prostatic cancer.

Table S1. Design of multigene panel.

Gene	Reference Sequence	Chr	Locus		Gene Uniformity
ATM	NM_000051.3	chr11	108117686	108236240	97.43%
PALB2	NM_024675.3	chr16	23614775	23649278	100%
MRE11A	NM_005591.3	chr11	94203632	94163157	100%
RAD50	NM_005732.3	chr5	131977865	131944422	100%
BARD1	NM_000465.3	chr2	215593395	215646238	100%
NBN	NM_002485.4	chr8	90990443	90982790	99.22%
BRIP1	NM_032043.2	chr17	59763192	59858371	96.28%
RAD51C	NM_058216.2	chr17	56809840	56787356	100%
RAD51D	NM_001142571.2	chr17	33434380	33446637	99.99%
STK11	NM_000455.4	chr19	1220575	1223176	94.69%
MSH2	NM_000251.2	chr2	47630326	47637516	99.99%
MLH1	NM_000249.3	chr3	37053497	37070428	100%
MSH6	NM_000179.2	chr2	48010368	48033795	99.49%
PMS2	NM_000535.6	chr7	6045518	6037059	100%
EPCAM	NM_002354.2	chr2	47596640	47606198	95.35%
MUTYH	NM_001128425.1	chr1	45798430	45800188	93.40%
RECQL1	NM_032941.2	chr12	21627770	21652509	91.39%
TP53	NM_000546.5	chr17	7576532	7578294	99.35%
PTEN	NM_000314.6	chr10	89711870	89725234	82.32%
CHEK2	NM_007194.3	chr22	29091693	29121117	98.88%
CDH1	NM_004360.4	chr16	68862072	68856133	98.34%
CDK4	NM_000075.3	chr12	58143232	58145130	100%
CDKN2A	NM_001195132.1	chr9	21970896	21968775	99.56%
SMAD4	NM_005359.5	chr18	48591788	48593562	100%
APC	NM_000038.5	chr5	112111321	112163708	100%

Custom Panel were designed with Ampliseq Designer V.7.0 (https://ampliseq.com/protected/startPage.action); features: 25 genes, 610 amplicons with size range 125–275 bp and size: 113.732 Kb; RefSeq, Reference Sequence; (https://www.ncbi.nlm.nih.gov/refseq/); chr, chromosome.

Table S2. Sequencing metrics by NGS Analysis.

Sample ID	Mapped Reads	On Target	Mean Depth	Uniformity
P1	265974	95.50%	405.3	98.27%
P2	257859	95.72%	393.9	98.29%
P3	361558	96.82%	580.4	97.34%
P4	365817	96.59%	587.4	97.26%
P5	415920	96.24%	669.1	97.43%
P6	319226	96.72%	514.7	97.34%
P7	402847	96.59%	645.9	97.51%
P8	314152	96.79%	506.7	97.43%
P9	384365	95.89%	613.1	97.49%
P10	390333	96.76%	625.2	97.13%
P11	421135	96.70%	678.9	97.19%
P12	408001	97.03%	655.0	97.53%
P13	327714	96.72%	532.2	97.02%
P14	296464	96.85%	483.6	97.00%
P15	309866	97.17%	510.4	96.89%
P16	296702	96.91%	485.2	97.02%

P17	321173	97.04%	524.5	97.07%
P18	323141	96.85%	528.0	96.98%
P19	253280	97.38%	415.7	96.91%
P20	356567	96.72%	579.8	97.08%
P21	295855	96.54%	481.8	96.85%
P22	279793	96.99%	457.5	97.25%
P23	270964	96.81%	442.8	96.98%
P24	309334	96.23%	506.7	97.41%
P25	289469	96.16%	470.5	97.51%
P26	317876	95.72%	518.3	97.43%
P27	277975	95.81%	453.0	97.58%
P28	295275	95.99%	481.1	97.37%
P29	302214	96.15%	493.9	97.45%
P30	305939	96.63%	502.1	97.44%
P31	270857	96.27%	444.0	97.59%
P32	303282	96.07%	495.7	97.13%
P33	287175	96.12%	469.8	97.52%
P34	278560	96.29%	456.2	97.45%
P35	291093	96.88%	477.9	97.92%
P36	308171	96.55%	505.4	97.33%
P37	272634	95.75%	444.7	97.47%
P38	285321	96.23%	467.6	97.75%
P39	307511	95.67%	499.5	97.84%
P40	248018	96.97%	406.6	98.29%
P41	240847	96.67%	393.5	98.37%
P42	252759	96.86%	414.8	98.29%
P43	265843	96.66%	434.7	98.32%
P44	277000	97.04%	454.4	97.75%
P45	272515	96.50%	446.1	98.24%
P46	252472	96.74%	413.6	98.24%
P47	268628	96.41%	438.6	98.62%
P48	255448	96.27%	417.0	98.25%
P49	277743	97.13%	456.1	98.02%
P50	256926	96.87%	422.6	98.36%
P51	247390	97.05%	409.0	98.02%
P52	242946	97.14%	399.6	98.00%
P53	244278	96.45%	399.3	98.01%
P54	273698	96.44%	447.6	98.38%
P55	288082	96.29%	470.2	98.27%
P56	256038	97.12%	421.1	98.21%
P57	265449	96.56%	378.6	96.96%
P58	261563	96.45%	374.6	97.17%
P59	286485	96.75%	410.9	97.45%
P60	238345	96.92%	341.3	96.42%
P61	238369	97.32%	342.6	96.21%
P62	243834	96.99%	349.0	96.56%
P63	258227	97.35%	371.6	96.95%
P64	265357	96.94%	379.7	96.96%
P65	284650	95.97%	403.6	97.19%
P66	258638	96.67%	369.2	96.40%

P67	281147	96.41%	400.9	97.12%
P68	240022	96.67%	344.0	96.78%
P69	245741	96.34%	350.1	97.13%
P70	328912	96.64%	538.6	97.08%
P71	306955	97.14%	506.5	96.68%
P72	285526	96.74%	467.6	96.73%
P73	276866	96.72%	453.1	97.18%
P74	308770	96.57%	507.7	97.34%
P75	274247	96.40%	449.4	97.44%
P76	284436	96.68%	466.8	97.42%
P77	299980	96.77%	493.7	97.27%
P78	309146	97.20%	511.9	96.87%
P79	312151	95.90%	506.9	97.37%
P80	360830	96.46%	593.7	97.72%
P81	312941	96.36%	514.1	97.00%
P82	331309	97.14%	549.7	97.73%
P83	295512	97.07%	487.1	96.33%
P84	358633	96.80%	591.4	97.15%
P85	341502	97.08%	563.0	97.09%
P86	323585	96.80%	534.9	96.53%
P87	303142	96.50%	499.8	96.08%
P88	324612	96.56%	533.7	96.54%
P89	179612	96.37%	291.8	98.05%
P90	224188	97.09%	366.5	98.26%
P91	211038	96.83%	343.1	97.80%
P92	210598	97.19%	345.2	97.95%
P93	215158	97.07%	351.2	97.46%
P94	233815	97.28%	383.3	98.25%
P95	269618	96.90%	440.9	98.18%
P96	213535	97.21%	348.3	97.91%
P97	234410	97.04%	381.9	98.12%
P98	274238	97.24%	451.6	97.83%
P99	238687	97.38%	392.6	97.19%
P100	245952	96.92%	402.4	97.15%
P101	242043	96.78%	394.0	98.29%
P102	243615	97.21%	399.7	96.66%
P103	268001	97.25%	439.2	97.09%
P104	236380	93.96%	371.7	97.90%
P105	240343	96.50%	390.6	96.59%
P106	231792	92.98%	362.6	97.31%
P107	235762	90.30%	358.3	96.74%
P108	237846	88.19%	352.2	96.85%
P109	352315	96.94%	575.8	97.22%
P110	248011	97.34%	410.8	98.01%
P111	228032	97.39%	378.8	97.26%
P112	252954	96.75%	417.7	97.51%
P113	372372	97.25%	613.4	98.50%

The distribution of average coverage depth for all exon targets, displayed by sample. ROI, Regions of interest.

Table S3. Clinical Characteristics of probands with VUS variants.

ID Sample	Sex	Gene	VUS	Age (Years)	Age at Onset (Years)	Tumour Site	histological diagnosis	Grading	Other Personal History of Cancer	Family History of Cancer (*n* of 1st and 2nd degree affected relatives)
P108	F	*ATM*	c.655T>C, p.(Cys219Arg)	54	47	ovary	HSGC	na	-	BC (3); UC (1)
P80	F	*ATM*	c.4564G>A, p.(Gly1522Ser)	44	42	breast (left)	IDC	G2	-	BC (1 + 1); CC (2)
P03	F	*ATM*	c.5882A>G, p.(Tyr1961Cys)	58	46	breast (left)	IDC	G2	-	BC (3); GI (4 + 1); M (1); LC (1); unk (1); multi C (1)
P102	F	*ATM*	c.6976-4A>G, p.?	53	42	breast (unilat)	ILC	G2	-	BC (1); PC (1); PrC (3 + 1); Leuk(1); M (2); LC (1), KC (1); multi C (1)
P51	F	*ATM*	c.8066A>G, p.(Glu2689Gly)	67	45	breast (bil)	IDC	G1	-	-
P22	F	*BARD1*	c.211T>A, p.(Cys71Ser)	49	49	breast (sin)	IDC	G3	-	UC (1); M(1); CC (1); LC (1); unk (1); multi C (1)
P74	F	*BRIP1*	c.146G>A p.(Gly49Glu)	65	65	ovary	HSGC	na	-	BC (1 + 1); unk (1)
P109	F	*BRIP1*	c.356A>G, p.(Asn119Ser)	42	42	breast (right)	IDC	G1	-	BC (1); PrC (1); CC (1); CNS C(1)
P17	F	*PALB2*	c.3436C>A, p.(Gln1146Lys)	46	35	breast (left)	IDC	G3	-	UC (1); LC (1)
P31	F	*PALB2*	c.3436C>A, p.(Gln1146Lys)	64	62	ovary	HSGC	na	-	BC (1); BlC (1); CC (1)
P47	F	*RAD50*	c.2390G>A, p.(Arg797Lys)	51	48	breast (right)	IDC	G3	-	BC (1), OC (1); M (1)
P72	F	*STK11*	c.434A>G, p.(Glu145Gly)	53	52	breast (right)	IDC	G3	-	BC (4); CC (1); CNS (1); Leuk (1); unk (3)
P05	F	*EPCAM*	c.450C>G, p.(His150Gln)	59	57	breast (bil)	IDC	G2+G1	KC	BC (1 + 1); CC (1); PC (1)
P09	F	*MSH2*	c.1787A>G, p.(Asn596Ser)	57	54	breast (left)	IDC	G1	-	BC (1); unk (1)
P104	F	*MSH6*	c.2201T>A, p.(Val734Glu)	63	47	breast (left)	IDC	na	-	BC (3 + 1); GC (1)
P45	F	*PMS2*	c.1253C>T, p.(Ser418Phe)	39	39	breast (left)	IDC	G3	-	BC (1); UC (1); unk (1)
P62	M	*MUTYH*	c.505-12T>G, p.?	49	43	breast (left)	IDC	G1	-	BC (1 + 1); CC (1); multi C (1)
P19	F	*APC*	c.5283C>G, p.(Asn1761Lys)	67	unk	breast (unilat)	na	na	na	na

P103 *	F	*APC*	c.7667C>T, p.(Ser2556Leu)	55	55	breast (right)	IDC	G3	-	BC (1); Leuk (1)
P82	F	*CDH1*	c.674T>C, p.(Ile225Thr)	36	36	breast (right)	IDC	G3	-	BC (3)
P86	F	*CDKN2A*	c.415G>A, p.(Gly139Ser)	46	46	breast (left)	IDC	G1	-	BC (2 + 1); PrC (1); CNS (2); CC (1)
P70	F	*CHEK2*	c.444+3A>G, p.?	42	42	breast (right)	IDC	G1	-	BC (2 + 1); M (2); CC (1); BlC (1)
P78	F	*CHEK2*	c.715G>A, p.(Glu239Lys)	46	38	breast (right)	IDC	G3	M	OC (1); UC (1)

Abbreviations: IDC, infiltrating ductal carcinoma; infiltrating lobular carcinoma; HGSC, high-grade serous carcinoma; BC, breast cancer; OC, ovarian cancer; PrC, prostatic cancer; CNSC, central nervous system cancer; LC, lung cancer; M, Melanoma; GC, gastric cancer; NHL, non-Hodgkin lymphoma; RC, renal cancer; CC, Colon Cancer; EC, esophageal cancer; TC, tyroid cancer; KC, kidney cancer; PC, pancreatic cancer; S, sarcoma; BlC, bladder cancer; UC, uterine cancer; HNC, head-neck carcinoma; dML, diffuse mesenteric leyomiomatosis; unk C, cancer at not specified site. * In patient P103 we detected also c.4703A>G p.(His1568Arg) variant in *ATM* gene; p.?, consequence on protein structure unknown.

Table S4. Females with breast cancer (fBC).

**Characteristics**	**P/LP pts (*n* = 8)**	**NI pts (*n* = 79)**	**N pts (*n* = 60)**	**VUS pts (*n* = 19)**	***p* Value**
**Age**					
at study	51 ± 8.65 (39–66)	54.73 ± 10.42 (836–83)	55.85 ± 10.63 (38–83)	51.20 ± 9.09 (36–67)	ns
Onset	47.37 ± 9.53 (35–64)	49.45 ± 10.48 (30–82)	50.77 ± 11.18 (30–82)	45.27 ± 6.44 (35–57)	ns
BC ≤ 40 years	25% (2/8)	17.72% (14/79)	16.67% (10/60)	21.05% (4/19)	ns
**Tumour type**					
CDI	62.5% (5/8)	83.33% (60/72)	79.63 (43/54)	94% (17/18)	ns
Cdis	25% (2/8)	4.17% (3/72)	5.56% (3/54)	0% (0/18)	ns
CLI	0% (0/8)	6.94% (5/72)	7.41% (4/54)	5.55% (1/18)	ns
Clis	0% (0/8)	0% (0/72)	0% (0/54)	0% (0/18)	ns
mix	12.5% (1/8)	5.55% (4/72)	7.41% (4/54)	0% (0/18)	ns
bilateral	62.5% (5/8)	16.49% (13/79)	18.33% (11/60)	10.53% (2/19)	**<0.05**
other cancers	25% (2/8)	8.86% (7/79)	8.33% (5/60)	10.52% (2/19)	ns
**Tumour grade**					
G1-G2	57.14% (4/7)	67.19% (43/64)	66.67% (32/49ì8)	68.75% (11/16)	ns
G3	42.86% (3/7)	32.81% (21/64)	33.33% (16/48)	31.25% (5/16)	ns
**Tumour stage**					
Stage 0	40% (2/5)	9.30% (4/43)	10.71% (3/28)	6.66% (1/15)	ns

Stage 1	40% (2/5)	53.49% ((23/43)	50% (14/28)	60% (9/15)	ns
Stage 2	0% (0/5)	27.90% (12/43)	28.57% (8/28)	26.67% (4/15)	ns
Stage 3–4	20% (1/5)	9.30% (4/43)	10.71% (3/28)	6.66% (1/15)	ns
MIB-1 ≥20	25% (2/8)	44.83% (26/58)	41.86% (18/43)	53.33 (8/15)	ns
**Receptor status**					
HR+ Her2−	71.43% (5/7)	32.75% (19/58)	60.89% (28/46)	50% (7/14)	ns
HR+ Her2+	14.28% (1/7)	58.62% (34/58)	26.09% (12/46)	42.86% (6/14)	<0.05 (P/LP vs. NI)
HR− Her2+	14.28% (1/7)	3.45% (2/58)	4.35% (2/46)	0% (0/14)	ns
HR− Her2−	14.28% (1/7)	5.17% (3/58)	6.52% (3/46)	7.14% (1/14)	ns
**Familiarity for cancer**					
Familiarity	100% (8/8)	98.67% (74/75)	100% (57/57)	94.44% (17/18)	ns
BC	50% (4/8)	85.33% (64/75)	87.72% (50/57)	77.78% (14/18)	<0.05 (P/LP vs. NI; P/LP vs. NM)
OC	12.5% (1/8)	22.67% (17/75)	26.31% (15/57)	11.11% (2/18)	ns
Pr	50% (4/8)	24% (18/75)	26.31% (15/57)	16.67% (3/18)	ns
P	0% (0/8)	10.66% (8/75)	10.53% (6/57)	11.11% (2/18)	ns
other C	62.5% (5/8)	72% (54/75)	68.42% (39/57)	83.33% (15/18)	ns
multi C	25% (2/8)	16% (12/75)	15.79% (9/57)	16.67% (3/18)	ns

Comparison of clinical, histologic and familial characteristics between females with breast cancer carrying or not carrying pathogenic/ likely pathogenic variants in DDR genes.

Table S5. Patients with ovarian cancer.

Characteristics	P/LP pts (*n*= 3)	NI pts (*n*= 7)	NM (*n*= 4)	VUS (*n*= 3)	*p* Value
**Age**					
at study	48.67 ± 3.79	62.43 ± 7.39	63.5 ± 9	61 ± 6.08	<0.05
Onset	46.67 ± 4.04	53.85 ± 15.66	50.75 ± 19.96	58 ± 9.64	ns
**other cancers**					
	33.33% (1/3)	0% (0/7)	0% (0/4)	0% (0/3)	ns
**Familiarity for cancer**					
Familiarity	100% (3/3)	100% (7/7)	100% (4/4)	100% (3/3)	ns
BC	0% (0/3)	87.71% (6/7)	75% (3/4)	100% (3/3)	<0.05 (P/LP vs. NI)
OC	66.67% (2/3)	14.29% (1/7)	25% (1/4)	0% (0/3)	ns
PrC	66.67% (2/3)	0% (0/7)	0% (0/4)	0% (0/3)	0.0667
PC	0% (0/3)	0% (0/7)	0% (0/4)	0% (0/3)	ns
other C	100% (3/3)	71.43% (5/7)	50% (2/4)	100% (3/3)	ns
multi C	33.33% (1/3)	0% (0/7)	0% (0/4)	0% (0/3)	ns

Comparison of clinical and familial characteristics between probands with ovarian cancer carrying or not carrying pathogenic/likely pathogenic variants in DDR genes.

Table S6. Males with breast cancer (mBC).

Characteristics	P/LP pts (*n*= 2)	NI pts (*n* = 5)	N (*n*= 4)	VUS (*n* = 1)	*p* Value
**Age**					
at study	72	59.6 ± 12.42	62.25 ± 12.60	49	ns
Onset	65.5 ± 9.19	58.25 ± 15.66	63.33 ± 14.58	43	ns
**Tumor type**					
CDI	100% (2/2)	40% (2/5)	1	1	ns
other cancers	50% (1/2)	0% (0/5)	0	0	ns
**Familiarity for cancer**					
Familiarity	100% (2/2)	100% (4/4)	3/4	1	ns
BC	100% (2/2)	50% (2/4)	1/3	1	ns
OC	100% (2/2)	25% (1/4)	0/3	1	ns
PrC	100% (2/2)	25% (1/4)	1/3	0	ns
PC	0% (0/2)	0% (0/4)	0	0	ns

other C	100% (2/2)	100% (4/4)	3/3	1	ns
multi C	50% (1/2)	50% (2/4)	1/3	1	ns

Comparison of clinical and familial characteristics between males with breast cancer carrying or not carrying pathogenic/likely pathogenic variants in DDR genes.

Table S7. Probands enrolled for their positive family history.

**Characteristics**	**P/LP pts**	**NI**	**p Value (P/LP vs. NI)** #
	(*n*= 3)	(*n*= 4)	-
**Age**	49.67 ± 3.05 (47–53)	57.5 ± 10.38 (45–67)	ns
cancers	100% (3/3)	0% (0/4)	<0.05
**Familiarity**			
BC	100% (3/3)	50% (2/4)	ns
OC	0% (0/3)	50% (2/4)	ns
PrC	33.33% (1/3)	50% (2/4)	ns
PC	33.33% (1/3)	50% (2/4)	ns
other C	100% (3/3)	75% (3/4)	ns
multi C	33.33% (1/3)	50% (2/4)	ns

Comparison of clinical and familial characteristics between probands enrolled for their positive family history malignancies (breast, BC; ovarian, OC, pancreatic PC, ±other tumors), with or without a personal diagnosis of cancers other than BC, OC and PC, carrying or not carrying pathogenic/ likely pathogenic variants in DDR. # Comparison limited by the small number of subjects in the two groups.
